# Association Between Age at Puberty and Bone Accrual From 10 to 25 Years of Age

**DOI:** 10.1001/jamanetworkopen.2019.8918

**Published:** 2019-08-09

**Authors:** Ahmed Elhakeem, Monika Frysz, Kate Tilling, Jon H. Tobias, Deborah A. Lawlor

**Affiliations:** 1 Medical Research Council Integrative Epidemiology Unit at University of Bristol, Population Health Sciences, Bristol Medical School, Bristol, United Kingdom; 2 Population Health Sciences, Bristol Medical School, University of Bristol, Bristol, United Kingdom; 3 Musculoskeletal Research Unit, Translational Health Sciences, Bristol Medical School, University of Bristol, Bristol, United Kingdom

## Abstract

**Importance:**

Bone health in early life is thought to influence the risk of osteoporosis in later life.

**Objective:**

To examine whether puberty timing is associated with bone mineral density accrual up to adulthood.

**Design, Setting, and Participants:**

This cohort study used data from the Avon Longitudinal Study of Parents and Children, a prospective population-based birth cohort initiated in 1991 to 1992 in southwest England. The participants were 6389 healthy British people who underwent regular follow-up, including up to 6 repeated bone density scans from ages 10 to 25 years. Data analysis was performed from June 2018 to June 2019.

**Exposures:**

Age at puberty from estimated age at peak height velocity (years).

**Main Outcomes and Measures:**

Gains per year in whole-body bone mineral density (grams per square centimeter), assessed by dual-energy x-ray absorptiometry at ages 10, 12, 14, 16, 18, and 25 years and modeled using linear splines.

**Results:**

A total of 6389 participants (3196 [50.0%] female) were included. The mean (SD) age at peak height velocity was 13.5 (0.9) years for male participants and 11.6 (0.8) years for female participants. Male participants gained bone mineral density at faster rates than did female participants, with the greatest gains in both male participants (0.139 g/cm^2^/y; 95% CI, 0.127-0.151 g/cm^2^/y) and female participants (0.106 g/cm^2^/y; 95% CI, 0.098-0.114 g/cm^2^/y) observed between the year before and 2 years after peak height velocity. When aligned by chronological age, per 1-year older age at puberty was associated with faster subsequent gains in bone mineral density; the magnitudes of faster gains were greatest between ages 14 and 16 years in both male participants (0.013 g/cm^2^/y; 95% CI, 0.011-0.015 g/cm^2^/y) and female participants (0.014 g/cm^2^/y; 95% CI, 0.014-0.015 g/cm^2^/y), were greater in male participants (0.011 g/cm^2^/y; 95% CI, 0.010-0.013 g/cm^2^/y) than in female participants (0.003 g/cm^2^/y; 95% CI, 0.003-0.004 g/cm^2^/y) between ages 16 and 18 years, and were least in both male participants (0.002 g/cm^2^/y; 95% CI, 0.001-0.003 g/cm^2^/y) and female participants (0.000 g/cm^2^/y; 95% CI, −0.001 to 0.000 g/cm^2^/y) between ages 18 and 25 years. Despite faster gains, older age at puberty was associated with persistently lower bone mineral density, changing from 0.050 g/cm^2^ (95% CI, −0.056 to −0.045 g/cm^2^) lower at age 14 years to 0.047 g/cm^2^ (95% CI, −0.051 to −0.043 g/cm^2^) lower at age 25 years in male participants and from 0.044 g/cm^2^ (95% CI, −0.046 to −0.041 g/cm^2^) to 0.034 g/cm^2^ (95% CI, −0.036 to −0.032 g/cm^2^) lower at the same ages in female participants.

**Conclusions and Relevance:**

People with older pubertal age should be advised on how to maximize bone mineral density and minimize its decrease in later life to help prevent fracture and osteoporosis.

## Key Points

**Question:**

Is puberty timing associated with growth-related bone accrual up to adulthood?

**Findings:**

In this cohort study of 6389 participants who underwent repeated bone density scans from ages 10 to 25 years, later puberty was associated with persistently lower bone mineral density, despite some catch-up during puberty.

**Meaning:**

People with older pubertal age should be advised on how to maximize bone density and minimize its decrease in later life to help prevent fracture and osteoporosis.

## Teaser

This cohort study of healthy individuals in England who underwent repeated bone density scans from ages 10 to 25 years describes the association between the timing of puberty and bone mineral density.

## Introduction

Peak bone mass at the end of growth is thought to be an important determinant of later-life risk of fracture and osteoporosis,^1,2,3,4,5^ a bone loss disorder with substantial and increasing health costs.^6,7^ For example, bone remodeling simulations showed that a 10% increase in peak bone mineral density (BMD) would delay osteoporosis by 13 years.^4^ Puberty is a key early life milestone that is characterized by endocrine-initiated reproductive maturation and a dramatic skeletal growth spurt in height.^8,9^ Although bone mass potential and puberty timing are both strongly heritable,^10,11,12^ evidence indicates that later puberty may lead to lower BMD in adolescence and adulthood^13,14,15,16^ and, thus, an increased risk of osteoporosis later in life. However, the association between puberty timing and long-term bone accrual from early life up to adulthood, including the extent and duration of any catch-up bone accrual by later maturing adolescents, has not been described, to our knowledge. Age at peak height velocity (APHV) is an accurate and precise marker of puberty timing that allows direct comparisons of this association between male and female individuals.^13,17^ Yet, to our knowledge, only 2 studies, which included 230 participants who underwent repeated bone scans^18^ and 500 male participants assessed once at baseline and once at follow-up,^19^ have examined the association between APHV and bone accrual.

Therefore, the aim of this study was to examine the association between age at puberty (measured by APHV) and bone accrual from ages 10 to 25 years in a large birth cohort of male and female participants. We assessed the rates of BMD and bone mineral content (BMC) accrual from before puberty up to adulthood relative to pubertal age and examined the association between age at puberty and subsequent BMD and BMC accrual. Our secondary aim was to examine the associations between age at puberty and site-specific bone accrual.

## Methods

### Study Sample

The Avon Longitudinal Study of Parents and Children (ALSPAC)^20,21^ is a prospective birth cohort study that recruited all pregnant women residing within the catchment area of 3 National Health Service authorities in southwest England with an expected date of delivery between April 1991 and December 1992. In total, 15 247 eligible pregnancies were enrolled in ALSPAC (75% response), resulting in 14 973 live births, of whom 14 899 were alive at 1 year of age. Detailed information has been collected from offspring and parents using questionnaires, data extraction from medical records, linkage to health records, and dedicated clinic assessments up to the last completed contact in 2018. Details of all available data can be found in the ALSPAC study website,^22^ which includes a fully searchable data dictionary and variable search tool. Ethics approval was obtained from the ALSPAC law and ethics committee and the local National Health Service research ethics committee. Written informed consent was obtained from all participants. This study followed the Strengthening the Reporting of Observational Studies in Epidemiology (STROBE) reporting guideline.

### Height Measurements

Numerous height measures have been obtained from various sources from birth to age 25 years, including from routine data collected from midwives, health visitors, linkage to child health records, and ALSPAC research clinic visits. Height from research clinic visits (annually or more frequently up to age 14 years and then at mean ages of 16, 18, and 25 years) was measured by accredited fieldworkers to the nearest 0.1 cm using a Harpenden stadiometer (Holtain Ltd). Measured heights were supplemented by extensive maternal and self-reported height records collected throughout the study. For these analyses, we restricted APHV estimation to individuals with height measurements taken between ages 5 and 20 years and at least one height measurement after age 9 years because these were relevant to assessment of APHV.

### Dual-Energy X-ray Absorptiometry Scans

All participants were invited to undergo whole-body dual-energy x-ray absorptiometry (DXA) scans as part of clinic assessments at mean ages 10, 12, 14, 16, 18, and 25 years. Scans were performed using a Lunar Prodigy scanner (Lunar Radiation Corp) and analyzed according to the manufacturer’s standard scanning software and positioning protocols. Scans were reanalyzed as necessary to ensure optimal placement of borders between adjacent subregions, and scans with anomalies were excluded.^23,24^ For our primary outcomes, we extracted whole-body (except for the head) BMD (grams per square centimeters) and BMC (grams) at each age. Our secondary outcomes were site-specific BMD and BMC (arms, legs, trunk, ribs, spine, and pelvis) from whole-body DXA, in addition to total hip and femoral neck BMD from up to 3 repeated hip DXA scans performed at ages 14, 18, and 25 years.

### Confounding Variables

Birth weight, ethnicity, socioeconomic position, body mass index (calculated as the weight in kilograms divided by height in meters squared), and diet in early life were hypothesized to potentially confound associations between puberty timing and bone outcomes and were included as model adjustments. Birth weights were recorded to the nearest gram and were extracted from hospital records. Ethnicity was based on the mother’s ethnic background and was reported during a general prenatal questionnaire. Early life socioeconomic position was based on the mother’s highest educational qualifications reported in pregnancy. Body mass index was derived from height and weight measurements taken at the age 7 years research clinic visit. Dietary intake in early life was based on the child’s daily energy intake (kilojoules per day), which was derived from food frequency questionnaires completed by the parents when the child was aged 7 years.^25^

### Statistical Analysis

Data analysis was performed from June 2018, to June 2019. Statistical analyses were performed in 2 stages. The first stage involved measuring age at puberty by estimating APHV, which was done using Superimposition by Translation and Rotation (SITAR) growth curve analysis.^13,26^ The SITAR models reduce complex growth data into clinically relevant parameters that represent the timing, intensity, and duration of the pubertal growth spurt, which, in turn, simplifies comparison between individuals and between male and female participants. We estimated APHV through transformation of the random age intercept that reflects individual differences in the timing of the growth spurt.

The second stage involved investigating the association between age at puberty and whole-body and site-specific BMD and BMC accrual up to adulthood using 2 sets of mixed-effects linear spline regression models.^27,28^ These models summarize nonlinear change through a series of linear splines joined at knot points by estimating person-specific rates of accrual during each growth period.^27,28^ We included APHV-by-age splines interaction terms to investigate whether bone accrual differs by age at puberty, and models with sex-by-age splines interaction terms were used to test differences in bone accrual between male and female participants.

In the first set of these analyses, we examined gains in BMD and BMC from childhood (prepuberty) to adulthood relative to pubertal age by centering chronological age at APHV. Results from these models can be interpreted as rates of BMD and BMC accrual during 4 periods: 8 years up to 1 year before APHV, 1 year before APHV to 2 years after APHV, 2 to 4 years after APHV, and 4 to 16 years after APHV. The estimated trajectories were plotted for male and female participants. In the second set of analyses, we excluded bone scans taken before APHV to investigate the associations between age at puberty and subsequent bone accrual up to adulthood. Results from these models can be interpreted as rate of BMD and BMC accrual per older age at puberty during 4 periods; up to age 14 years, 14 to 16 years, 16 to 18 years, and 18 to 25 years. The estimated trajectories from these models were plotted for individuals in the 10th, 50th, and 90th sex-specific APHV percentiles. We assessed whether adolescent differences in BMD and BMC according to pubertal age persisted into adulthood by regressing the estimated bone values at age 25 years on APHV.

We fitted initial unadjusted linear spline models followed by models that included adjustment for birth weight, ethnicity, maternal education, body mass index, and diet; unadjusted and adjusted estimates were similar, and only the adjusted estimates are presented. Participants were excluded from the linear spline analyses if they did not have a valid DXA scan from at least 1 age or complete data on pubertal age and confounders. In sensitivity analyses, the linear spline models were refitted after (1) reestimating APHV from measured heights only (ie, excluding any parental- or self-report of height)^29^ and (2) using age at menarche (reported prospectively by the mother in years and months) instead of APHV as a marker of pubertal age in female participants. All analyses were performed in R statistical software version 3.5.1 (R Project for Statistical Computing). We fitted SITAR models with the sitar package and linear spline models with the nlme package. A more detailed description of the statistical methods is provided in eMethods, eTable 1, and eTable 2 in the Supplement.

## Results

### Participant Characteristics

**Figure 1.**
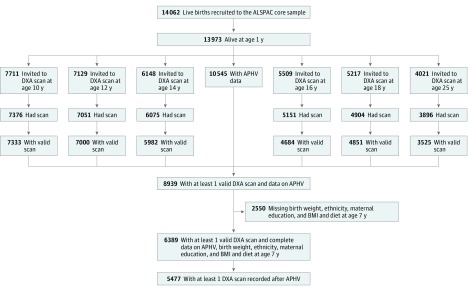
Study Flowchart for the Avon Longitudinal Study of Parents and Children (ALSPAC), 1991 to 2018 APHV indicates age at peak height velocity; BMI, body mass index; and DXA, dual-energy x-ray absorptiometry.

**Figure 2.**
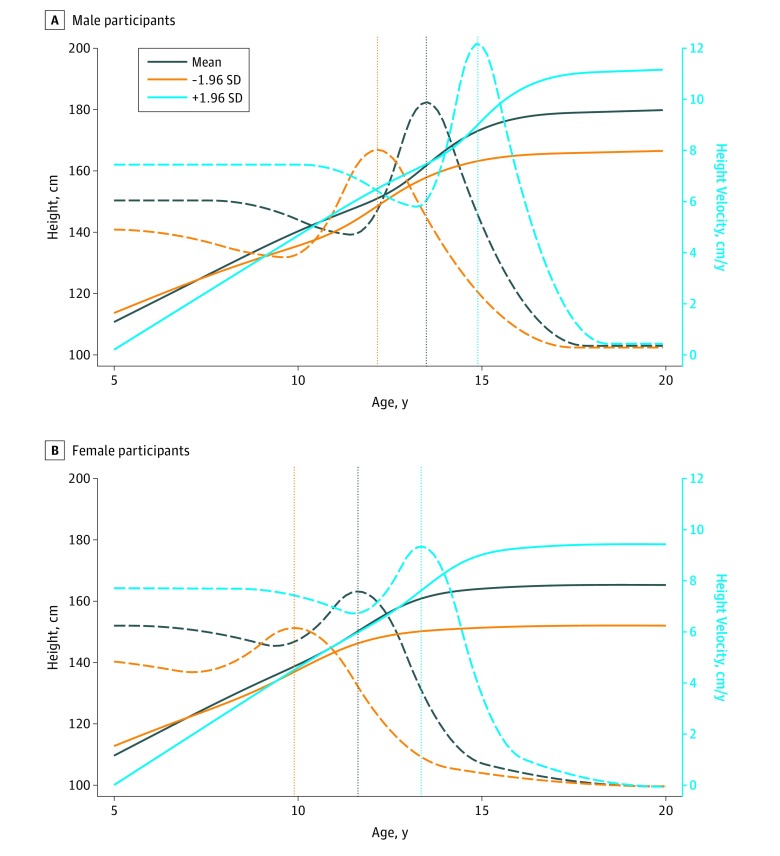
Height Growth and Height Velocity Curves Mean height growth curves (solid lines), mean height velocity curves (dashed lines), and mean age at peak height velocity (plus estimated curves and age at peak height velocity at 1.96 SD and −1.96 SD) (dotted vertical lines) are shown for male (A) and female (B) participants included in the Superimposition by Translation and Rotation models.

A total of 6389 participants (3196 [50.0%] female) had bone measures from at least 1 age (26 202 BMD or BMC measures in total; median [interquartile range] measurements per individual, 4 [2-6]), and data on APHV, birth weight, ethnicity, maternal education, and childhood body mass index and diet (Figure 1). Of these, 5477 participants (2975 female [54.3%]) had bone measurements from at least 1 age after peak height velocity (Figure 1). The numbers of participants with data from each age are presented in Figure 1 and in a footnote to the Table. Participants with missing data on covariates (28% of those potentially eligible) were excluded from the analyses. The APHV occurred around 2 years earlier in female participants (mean [SD], 11.6 [0.8] years) than male participants (mean [SD], 13.5 [0.9] years) (Table and Figure 2). The BMD and BMC increased over follow-up; their values were similar between male and female participants at assessment ages 10, 12, and 14 years and were higher in male participants at ages 16, 18, and 25 years (Table and eFigure 1 in the Supplement).

**Table.** Characteristics of Participants From the Avon Longitudinal Study of Parents and Children With Relevant Data, 1991 to 2018^a^

Characteristic	Mean (SD)	
	Male Participants (n = 3193)	Female Participants (n = 3196)
Age at peak height velocity, y	13.5 (0.9)	11.6 (0.8)
Age at dual-energy x-ray absorptiometry scan, y		
10	9.8 (0.3)	9.8 (0.3)
12	11.7 (0.2)	11.7 (0.2)
14	13.8 (0.2)	13.8 (0.2)
16	15.4 (0.3)	15.4 (0.3)
18	17.8 (0.4)	17.8 (0.4)
25	24.5 (0.8)	24.4 (0.8)
Whole-body bone mineral density, g/cm^2^, by age at assessment, y		
10	0.78 (0.1)	0.77 (0.1)
12	0.85 (0.1)	0.85 (0.1)
14	0.95 (0.1)	0.96 (0.1)
16	1.05 (0.1)	1.00 (0.1)
18	1.14 (0.1)	1.04 (0.1)
25	1.31 (0.1)	1.19 (0.1)
Whole-body bone mineral content, g, by age at assessment, y		
10	905.0 (174.6)	880.0 (191.1)
12	1186.1 (250.6)	1239.5 (298.4)
14	1721.3 (405.1)	1722.2 (343.1)
16	2226.5 (449.7)	1921.3 (344.8)
18	2562.8 (467.3)	2043.8 (373.2)
25	3047.7 (407.0)	2341.3 (274.7)
Birth weight, g	3474.2 (574.0)	3375.8 (506.3)
Race, No. (%)		
White	3137 (98.3)	3136 (98.1)
Nonwhite	56 (1.8)	60 (1.9)
Maternal education, No. (%)		
Certificate of secondary education	362 (11.3)	386 (12.1)
Vocational	282 (8.8)	256 (8.0)
General certificate of education, ordinary level	1160 (36.3)	1107 (34.6)
General certificate of education, advanced level	862 (27.0)	891 (27.8)
Degree or higher	527 (16.5)	556 (17.4)
BMI at age 7 y	16.1 (1.9)	16.3 (2.1)
Daily energy intake at age 7 y, kJ/d	7785.5 (1878.8)	7477.7 (1759.8)

Abbreviation: BMI, body mass index (calculated as the weight in kilograms divided by height in meters squared).

^a^ Numbers of participants at each age: 10 years, 2839 male and 2864 female; 12 years, 2691 male and 2730 female; 14 years, 2360 male and 2440 female; 16 years, 1791 male and 2000 female; 18 years, 1674 male and 2072 female; and 25 years, 1056 male and 1685 female. Numbers of participants at each age after removing bone measures recorded before age at peak height velocity: 10 years, 0 male and 57 female; 12 years, 55 male and 1518 female; 14 years, 1528 male and 2474 female; 16 years, 1741 male and 2036 female; 18 years, 1674 male and 2116 female; and 25 years, 1056 male and 1725 female.

### BMD and BMC Accrual From Childhood (Prepuberty) to Adulthood

**Figure 3.**
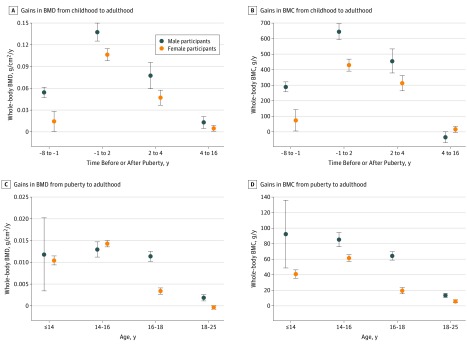
Gains in Bone Mineral Density (BMD) and Bone Mineral Content (BMC) Gains in BMD and BMC are shown from childhood (prepuberty) to adulthood, with age centered at pubertal age (ie, age at peak height velocity [APHV]) (A, BMD; B, BMC) and from puberty to adulthood per 1-year older age at puberty (C, BMD; D, BMC). Circles denote means, and whiskers denote 95% confidence intervals. Estimates were adjusted for birth weight, ethnicity, maternal education, and childhood body mass index and dietary intake. These estimates are presented in eTable 3 in the Supplement.

**Figure 4.**
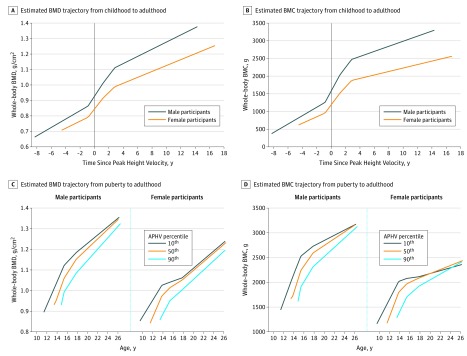
Estimated Bone Mineral Density (BMD) and Bone Mineral Content (BMC) Trajectories Mean estimated BMD and BMC trajectories are shown from childhood (prepuberty) to adulthood (A, BMD; B, BMC) against time since puberty (ie, age at peak height velocity [APHV], represented by vertical line), and from puberty to adulthood for individuals in the 10th (<12.4 years in male and <10.6 years in female participants), 50th (13.3-13.5 years in male and 11.4-11.6 years in female participants), and 90th (>14.5 years in male and >12.7 years in female participants) pubertal age (APHV) percentiles (C, BMD; D, BMC).

Figure 3A and B presents the rates of whole-body BMD and BMC accrual relative to pubertal age (APHV) during each growth period from childhood (prepuberty) to adulthood. Both male and female participants had gains in BMD during all 4 growth periods (ie, from 8 years before and up to 16 years after age of peak height growth) and gains in BMC during the first 3 periods (ie, up to 4 years after APHV). Male participants gained BMD and BMC at faster rates than did female participants from childhood and up to 4 years after APHV. The fastest gains in BMD and BMC in both male and female participants were between the year before APHV and up to 2 years after APHV (0.139 g/cm^2^/y [95% CI, 0.127-0.151 g/cm^2^/y] for male participants vs 0.106 g/cm^2^/y [95% CI, 0.098-0.114 g/cm^2^/y] for female participants). No further gains in BMC in either male or female participants were observed from 4 years after APHV, with some evidence of modest loss in BMC in male participants during this period (eTable 3 in the Supplement). Figure 4A and B shows the mean estimated trajectories from childhood to adulthood for male and female participants, which illustrate these rapid pubertal gains in BMD and BMC.

### Age at Puberty and Subsequent BMD and BMC Accrual

Figure 3C and D shows the rates of whole-body BMD and BMC accrual per 1-year older age at puberty (ie, APHV) during each growth period from the time of peak height velocity up to adulthood. Older age at puberty was associated with faster gains in BMD and BMC in both male and female participants. Gains in BMD continued at a similar pace up to age 18 years in male participants (up to age 14 years, 0.012 g/cm^2^/y [95% CI, 0.003-0.020 g/cm^2^/y]; between ages 14 and 16 years, 0.013 g/cm^2^/y [95% CI, 0.011-0.015 g/cm^2^/y]; between ages 16 and 18 years, 0.011 g/cm^2^/y [95% CI, 0.010-0.013 g/cm^2^/y]) but had slowed considerably between ages 16 and 18 years in female participants (up to age 14 years, 0.010 g/cm^2^/y [95% CI, 0.009-0.011 g/cm^2^/y]; between ages 14 and 16 years, 0.014 g/cm^2^/y [95% CI, 0.014-0.015 g/cm^2^/y]; between ages 16 and 18 years, 0.003 g/cm^2^/y [95% CI, 0.003-0.004 g/cm^2^/y]). Between ages 18 and 25 years, BMD gains had slowed substantially in the male participants (0.002 g/cm^2^/y; 95% CI, 0.001-0.003 g/cm^2^/y) and had ceased completely in the female participants (0.000 g/cm^2^/y; 95% CI, −0.001 to 0.000 g/cm^2^/y) with older pubertal age. Gains in BMC in male participants continued at similar peak levels up to age 16 years, somewhat slowing between ages 16 and 18 years before a more substantial decrease in pace between 18 and 25 years. In female participants, the gains in BMC peaked between 14 and 16 years and then continued slowing considerably between ages 16 to 18 and 18 to 25 years.

Examining associations between age at puberty and estimated bone values showed evidence of persisting differences in BMD and of catch-up in BMC by age 25 years. For example, in male and female participants, respectively, per 1-year older APHV was associated with 0.050 g/cm^2^ (95% CI, −0.056 to −0.045 g/cm^2^) and 0.044 g/cm^2^ (95% CI, −0.046 to −0.041 g/cm^2^) lower BMD at age 14 years and with 0.047 g/cm^2^ (95% CI, −0.051 to −0.043 g/cm^2^) and 0.034 g/cm^2 ^(95% CI, −0.036 to −0.032 g/cm^2^) lower BMD at age 25 years. In contrast, in the male and female participants combined, per 1-year older APHV was associated with 185.5 g (95% CI, −196.4 to −174.6 g) lower BMC at age 14 years and with 116.7 g (95% CI, 111.7-121.8 g) higher BMC at age 25 years.

Figure 4C and D shows the mean estimated BMD and BMC trajectories for individuals in the 10th, 50th, and 90th APHV percentiles. The oldest pubertal age group in both sexes (ie, the 90th percentile of APHV) gained BMD and BMC at faster rates over follow-up, which partially reduced the differences in BMD and more substantially reduced differences in BMC between groups by age 25 years. Assessing associations with estimated bone values at age 25 years showed that male and female participants in the oldest pubertal age group had 0.054 g/cm^2^ (95% CI, −0.60 to −0.49 g/cm^2^) lower BMD (equivalent to 0.5-SD difference in BMD) and 90.9 g (95% CI, 79.6-102.1 g) higher BMC when compared with those in the youngest pubertal age group. eFigure 2 in the Supplement shows that the trajectories in Figure 4C and D were largely unchanged when APHV was estimated from measured heights only. eFigure 3 in the Supplement shows the estimated trajectories for age at menarche percentile groups in female participants, which were very similar to the trajectories for APHV percentile groups presented in Figure 4C and D.

### Age at Puberty and Site-Specific Bone Trajectories

eFigures 4 to 9 in the Supplement show the mean estimated BMD and BMC trajectories for arms, legs, trunk, spine, ribs, and pelvis, respectively, in male and female participants in the 10th, 50th, and 90th APHV percentiles. Overall, these were similar to the main whole-body trajectories in that those in the oldest APHV groups started lower and exhibited some catch-up over follow-up. eFigure 10 in the Supplement shows similar mean estimated hip BMD trajectories. Male and female participants in the oldest APHV group had lower total hip and femoral neck BMD at age 14 years, and these differences were more substantially reduced in male than female participants at ages 18 and 25 years (eFigure 10 in the Supplement). However, the lack of hip measures from earlier age makes it difficult to discern whether these patterns are different from trajectories of whole-body parameters.

## Discussion

We investigated the association between age at puberty (measured by APHV) and bone accrual from ages 10 to 25 years in male and female participants from a large prospective British birth cohort. The BMD and BMC increased over follow-up with sex differences emerging during puberty. Male participants accrued BMD and BMC at faster rates than did female participants from before puberty and up to 4 years after APHV. The fastest gains in BMD and BMC in both male and female participants were during the year before APHV to 2 years after APHV. Older pubertal age was associated with an initially accelerating and subsequently decelerating faster BMD and BMC accrual over follow-up. This catch-up was greater in male participants than in female participants between ages 16 and 18 years for BMD and between ages 14 and 25 years for BMC. Despite these faster gains, older pubertal age remained associated with lower BMD throughout follow-up to age 25 years and with a BMC that was lower at younger ages but higher by age 25 years. Findings were overall similar for site-specific BMD and BMC accrual.

The findings that the greatest gains in BMD and BMC occurred during the year before and 2 years after APHV and that gains in BMC ceased 4 years after APHV agree with those of a recent study^30^ of the temporal association between peak height and peak bone accrual; however, that study did not test the association between APHV and bone accrual. That study,^30^ which included 2000 participants aged 5 to 19-years who underwent annual DXA scans for up to 7 years, showed that up to 36% of BMC is acquired in the 2 years before and 2 years after APHV. Our study expands on this by showing that, in contrast to BMC, gains in BMD continued into adulthood. We also showed that male participants had faster gains in both BMD and BMC from before puberty than did female participants but that sex differences in BMD and BMC emerged during puberty, which is consistent with the suggestion that sex hormones are driving sexual dimorphism in body composition.^31^

Our study contributes to previous research by documenting a process of transient pubertal catch-up in BMD and BMC among those with older pubertal age that did not fully eliminate differences in BMD. This agrees with findings that differences in BMD and BMC by APHV group were attenuated at follow-up in a mixed sample of 230 participants aged 8 to 14 years with long-term follow-up^18^ and in 500 male participants aged 18 years who were assessed at baseline and 5 years later.^19^ Our work adds to these studies by having considerably larger numbers and repeated measures of BMD from ages 10 to 25 years and by including female and male participants. This allowed us to demonstrate transient pubertal catch-up gains in BMD among those with older pubertal age, which were greater for male than for female participants. In contrast to BMD, we found that later puberty was associated with more prolonged catch-up in BMC, leading to higher total levels in adulthood. This finding may be explained by those reaching puberty later ultimately ending up taller^17,32^ and, thus, having bigger (albeit less dense) bones, but further research is required to disentangle the complex associations between pubertal growth and bone mineralization up to adulthood.

Given the persisting associations between later puberty and lower BMD reported here and evidence that peak bone mineralization lags behind peak height accrual,^30^ our findings suggest that adolescents who mature later may be at higher risk of fractures throughout adolescence.^15,19,30^ Because differences in BMD persisted up to age 25 years, including at the spine and hip sites, which are preferentially affected by osteoporotic fracture in later life,^33^ those with older pubertal age could also be at increased risk of osteoporosis in later life,^13^ although continued follow-up of this cohort is required to identify how associations might vary through adult life.

### Limitations

To our knowledge, this is the largest study on BMD in adolescents to date and has a longer follow-up than previous studies, but some limitations should be noted. Dual-energy x-ray absorptiometry is routinely used in clinical practice to assess BMD and osteoporosis; however, measurement error and changing soft-tissue distribution might influence findings. Newer imaging approaches could be used to investigate structural changes in bone microarchitecture, including growth-associated cortical porosity.^34^ Using APHV as measure of age at puberty is a key strength because it allows the same analyses in male and female participants and is less prone to differential measurement error than pubertal staging using reports of sexual maturity, such as Tanner stages. Measurement error, particularly from the self or parental reports of height, may have influenced our results. However, because parents would be unaware of their child’s subsequent bone measurements, that is unlikely to have been systematic. Furthermore, restricting analyses to research-clinic measures of height only produced similar results. In addition, similar results were obtained when using age at menarche as an alternative marker of pubertal age in female participants.

The linear spline models reduced bias resulting from missing bone data by including all participants with at least 1 bone measure under the missing-at-random assumption^27,28^ (ie, that the probability of missing bone data is assumed to be associated with model covariates and not associated with unmeasured factors). Although it is not possible to fully test that the assumption of missing at random holds, the probability of missing bone data among those included in the linear spline analysis was associated with the variables included in the models (eTable 1 in the Supplement). Those excluded from the analyses because of missing all 6 DXA scans were socioeconomically different from the analytic sample (eTable 2 in the Supplement), which might limit the generalizability of our findings. Participants with missing data on covariates (28% of those potentially eligible) were excluded from the analyses, which might introduce a bias if they had systematically different bone measurements; however, this seems unlikely because participants would not have known their bone measurements.^35,36^ Our study sample were mostly of white British ethnicity, which means that the findings may not be generalizable to participants of other ethnicities, with potentially different bone accrual rates.^30^ Also, our analyses were performed in 2 stages rather than as a single joint model, which might introduce a bias^37,38^; however, given the complex nonlinear SITAR models, joint modeling is unlikely to be feasible for this study.

## Conclusions

This large and long-running prospective follow-up cohort study showed that later puberty was associated with persistently lower BMD from ages 10 to 25 years in male and female participants, despite faster gains in BMD during puberty in those with older pubertal age. Future studies should seek more robust evidence of associations between puberty timing and bone accrual from large emerging collaborations such as the European Union’s Child Cohort Network,^39^ which can provide more repeated measures over longer follow-up in populations with differing confounding structures and with larger sample sizes that can support methods that are statistically inefficient, such as within sibship and mendelian randomization analyses.^40,41^ Our findings suggest that advice on how to increase and maintain BMD such as through physical activity^42,43^ should be offered to people with older pubertal age.

## Notes

Supplement. **eMethods.** Supplemental Methods. **eTable 1.** Comparison on Model Covariates Between Participants Included in the Linear Spline Models That Had All Six DXA Measures With Those Also Included in the Analysis But Were Missing at Least One DXA Measure **eTable 2.** Comparison on Model Covariates Between Those Included in the Linear Spline Models With Those That Were Excluded From the Analysis Due to Missing All Six DXA Measures, Despite Having Data on APHV **eReferences** **eTable 3.** Gains in BMD and BMC During Each Period (A) From Childhood (Prepuberty) to Adulthood, With Age Centered at Pubertal Age (ie, APHV) and (B) From Puberty to Adulthood Per 1-Year Older Age at Puberty **eFigure 1.** Observed Whole-body BMD and BMC at Each Follow-up Assessment From 10 to 25 Years **eFigure 2.** Predicted Mean Trajectories of Whole-body BMD and BMC From APHV to Adulthood for Individuals in the 10th, 50th, and 90th APHV Percentiles, Using APHV From Measured Heights **eFigure 3.** Predicted Mean Trajectories of Whole-body BMD and BMC From Menarche to Adulthood for Females in the 10th, 50th, and 90th Age at Menarche Percentiles **eFigure 4.** Predicted Mean Trajectories of Arms BMD and BMC From APHV to Adulthood for Individuals in the 10th, 50th, and 90th APHV Percentiles **eFigure 5.** Predicted Mean Trajectories of Legs BMD and BMC From APHV to Adulthood for Individuals in the 10th, 50th, and 90th APHV Percentiles **eFigure 6.** Predicted Mean Trajectories of Trunk BMD and BMC From APHV to Adulthood for Individuals in the 10th, 50th, and 90th APHV Percentiles **eFigure 7.** Predicted Mean Trajectories of Spine BMD and BMC From APHV to Adulthood for Individuals in the 10th, 50th, and 90th APHV Percentiles **eFigure 8.** Predicted Mean Trajectories of Ribs BMD and BMC From APHV to Adulthood for Individuals in the 10th, 50th, and 90th APHV Percentiles **eFigure 9.** Predicted Mean Trajectories of Pelvis BMD and BMC From APHV to Adulthood for Individuals in the 10th, 50th, and 90th APHV Percentiles **eFigure 10.** Predicted Mean Trajectories of Total Hip and Femur Neck BMD From APHV to Adulthood for Individuals in the 10th, 50th, and 90th APHV Percentiles
