# Supplementary material for: Association Between Age at Puberty and Bone Accrual From 10 to 25 Years of Age
Source: JAMA Netw Open. 2019 Aug 9;2(8):e198918. doi: 10.1001/jamanetworkopen.2019.8918 (PMC6692837; doi:10.1001/jamanetworkopen.2019.8918)
Supplement: Supplement. — eMethods. Supplemental Methods. eTable 1. Comparison on Model Covariates Between Participants Included in the Linear Spline Models That Had All Six DXA Measures With Those Also Included in the Analysis But Were Missing at Least One DXA Measure eTable 2. Comparison on Model Covariates Between Those Included in the Linear Spline Models With Those That Were Excluded From the Analysis Due to Missing All Six DXA Measures, Despite Having Data on APHV eReferences eTable 3. Gains in BMD and BMC During Each Period (A) From Childhood (Prepuberty) to Adulthood, With Age Centered at Pubertal Age (ie, APHV) and (B) From Puberty to Adulthood Per 1-Year Older Age at Puberty eFigure 1. Observed Whole-body BMD and BMC at Each Follow-up Assessment From 10 to 25 Years eFigure 2. Predicted Mean Trajectories of Whole-body BMD and BMC From APHV to Adulthood for Individuals in the 10th, 50th, and 90th APHV Percentiles, Using APHV From Measured Heights eFigure 3. Predicted Mean Trajectories of Whole-body BMD and BMC From Menarche to Adulthood for Females in the 10th, 50th, and 90th Age at Menarche Percentiles eFigure 4. Predicted Mean Trajectories of Arms BMD and BMC From APHV to Adulthood for Individuals in the 10th, 50th, and 90th APHV Percentiles eFigure 5. Predicted Mean Trajectories of Legs BMD and BMC From APHV to Adulthood for Individuals in the 10th, 50th, and 90th APHV Percentiles eFigure 6. Predicted Mean Trajectories of Trunk BMD and BMC From APHV to Adulthood for Individuals in the 10th, 50th, and 90th APHV Percentiles eFigure 7. Predicted Mean Trajectories of Spine BMD and BMC From APHV to Adulthood for Individuals in the 10th, 50th, and 90th APHV Percentiles eFigure 8. Predicted Mean Trajectories of Ribs BMD and BMC From APHV to Adulthood for Individuals in the 10th, 50th, and 90th APHV Percentiles eFigure 9. Predicted Mean Trajectories of Pelvis BMD and BMC From APHV to Adulthood for Individuals in the 10th, 50th, and 90th APHV Percentiles eFigure 10. Predicted Mean Trajecto [file jamanetwopen-2-e198918-s001.pdf]

Supplementary Online Content

Elhakeem A, Frysz M, Tilling K, Tobias JH, Lawlor DA. Association between age at puberty and bone accrual from 10 to 25 years of age. *JAMA Netw Open*. 2019;2(8):e198918. doi:10.1001/jamanetworkopen.2019.8918

eMethods. Supplemental Methods

eTable 1. Comparison on Model Covariates Between Participants Included in the Linear Spline Models That Had All Six DXA Measures With Those Also Included in the Analysis But Were Missing at Least One DXA Measure

eTable 2. Comparison on Model Covariates Between Those Included in the Linear Spline Models With Those That Were Excluded From the Analysis Due to Missing All Six DXA Measures, Despite Having Data on APHV

eReferences

eTable 3. Gains in BMD and BMC During Each Period (A) From Childhood (Prepuberty) to Adulthood, With Age Centered at Pubertal Age (ie, APHV) and (B) From Puberty to Adulthood Per 1-Year Older Age at Puberty

eFigure 1. Observed Whole-body BMD and BMC at Each Follow-up Assessment From 10 to 25 Years

eFigure 2. Predicted Mean Trajectories of Whole-body BMD and BMC From APHV to Adulthood for Indivudals in the 10th, 50th, and 90th APHV Percentiles, Using APHV From Measured Heights

eFigure 3. Predicted Mean Trajectories of Whole-body BMD and BMC From Menarche to Adulthood for Females in the 10th, 50th, and 90th Age at Menarche Percentiles

eFigure 4. Predicted Mean Trajectories of Arms BMD and BMC From APHV to Adulthood for Indivudals in the 10th, 50th, and 90th APHV Percentiles

eFigure 5. Predicted Mean Trajectories of Legs BMD and BMC From APHV to Adulthood for Indivudals in the 10th, 50th, and 90th APHV Percentiles

eFigure 6. Predicted Mean Trajectories of Trunk BMD and BMC From APHV to Adulthood for Indivudals in the 10th, 50th, and 90th APHV Percentiles

eFigure 7. Predicted Mean Trajectories of Spine BMD and BMC From APHV to Adulthood for Indivudals in the 10th, 50th, and 90th APHV Percentiles

eFigure 8. Predicted Mean Trajectories of Ribs BMD and BMC From APHV to Adulthood for Iindivudals in the 10th, 50th, and 90th APHV Percentiles

© 2019 Elhakeem A et al. *JAMA Network Open.*

eFigure 9. Predicted Mean Trajectories of Pelvis BMD and BMC From APHV to Adulthood for Indivudals in the 10th, 50th, and 90th APHV Percentiles

eFigure 10. Predicted Mean Trajectories of Total Hip and Femur Neck BMD From APHV to Adulthood for Indivudals in the 10th, 50th, and 90th APHV Percentiles

This supplementary material has been provided by the authors to give readers additional information about their work.

© 2019 Elhakeem A et al. *JAMA Network Open.*

eMethods. Supplemental Methods.

APHV was estimated using Superimposition by Translation and Rotation (SITAR) growth curve analysis (1, 2). SITAR is a shape invariant model consisting of a natural cubic spline mean curve of height vs age. SITAR fits this curve with three person-specific random-effects that reflect individual differences from the mean curve in size (random height intercept for person-specific shift up or down in the spline curve), velocity (random age scaling that reflects differences in the duration of the growth spurt in individuals) and tempo (random age intercept reflecting individual differences in the timing of the growth spurt). SITAR models were fitted separately for males and females with 5 degrees of freedom. APHV estimation was restricted to individuals with height measurements between ages 5 and 20 years and at least one height measure after age 9 years. Data cleaning similar to previous ALSPAC analyses was performed to remove data points with velocity exceeding 4 SDs and standardized residuals exceeding 4 in absolute value (2, 3, 4), leaving a total of 5223 males (with 55 752 height measurements) and 5322 females (with 59 318 height measurements) with an estimate of APHV. The final SITAR models explained 95.7% and 95.6% of variance in height in males and females respectively. APHV in years was estimated by transforming the random age intercept that reflects individual differences in the tempo (timing) of the growth spurt.

We subsequently investigated the relationship between APHV and whole-body BMD and BMC accrual using two sets of mixed-effects linear spline regression analyses. Linear spline models facilitate the summarising of nonlinear bone accrual through a series of linear splines joined at knot points (5, 6). They estimate mean and person-specific bone values at the first assessment and mean and person-specific rates of accrual during each of the different growth periods. Smoothing functions, prior subject knowledge and fit statistics were used to identify the best fitting models (5, 6) and subsequently knot point selection was based on the mean age at DXA scans. Models were fitted using maximum likelihood and included random intercepts and random age slopes. A 1st order autoregressive correlation structure was used to account for temporal autocorrelation, and a fixed variance structure was used to model heteroskedasticity. In both sets of linear spline models, we examined the relationship between APHV and rates of bone accrual by including APHV-by-age splines interaction terms and included sex-by-age splines interactions terms to test for statistical evidence of different bone trajectories for males and females. All models were adjusted for birth weight, ethnicity, maternal education and childhood BMI and energy intake. The linear spline models provided a good fit to the data, Pearson's correlations between the observed and predicted BMD and BMC values (without random effects) were 0.84 and 0.83.

© 2019 Elhakeem A et al. *JAMA Network Open.*

In the first analyses, age was centred at APHV in order to estimate rates of bone accrual relative to APHV; i.e. from before puberty up to adulthood. Knot points were placed at the mean ages of the 12, 14, and 16-year scans which equated to summarising rate of accrual from 7.7 up to 0.9 years prior to APHV, 0.9 year prior up to 2.1 years following APHV, 2.1 to 3.7 years following APHV, and 3.7 to 16 years after APHV. The mean predicted trajectories relative to APHV were plotted for males and females. For the second analyses, we excluded individuals with accrual from before APHV in order to investigate associations between APHV and subsequent rate of bone accrual up to adulthood. Here, knot points were placed at the mean ages of the 14 (centred at age 14), 16, and 18-year assessments, which then estimated associations with rate of accrual up to age 14, between 14–16, 16–18 and 18–25 years. To assess if difference in bone outcomes persisted into adulthood, predicted bone values at age 25 were regressed on APHV (after adjusting for predicted values at younger ages) and compared to associations with predicted values at age 14. To visualise associations between APHV and bone accrual, predicted mean trajectories were plotted for individuals in the 10th, 50th, and 90th sex-specific APHV percentiles.

In sensitivity analyses, we refitted models after re-estimating APHV from measured heights only (3) and using age at menarche (reported prospectively by the mother in years and months) instead of APHV as an alternative marker of age at puberty in females. For our secondary outcomes, we investigated associations between APHV and subsequent site-specific BMD and BMC accrual from whole-body DXA, and with total hip and femoral neck BMD accrual from hip DXA. Given that hips were only scanned at three different ages (14, 18 and 25), we fitted hip models using a single knot point placed at the mean ages of the 18-year assessment. The predicted mean trajectories were plotted for all secondary outcomes. All analyses were performed in R (R Foundation for Statistical Computing, Vienna); SITAR models were fitted with the ‘sitar’ package and linear spline models with the ‘nlme’ package.

The linear spline models reduced bias due to missing bone data by including all measures under the missing at random assumption (i.e. the probability of missing bone data assumed to be related to measured factors but unrelated to unmeasured factors). eTable 1 shows differences between those included in the analysis with complete and partial DXA data, and suggests that the probability of missing at least one bone measure was related to the model covariates, and thus possibly missing at random. Those missing all DXA measures were excluded from the analysis. eTable 2 shows differences between those included in the linear spline models and excluded due to missing all DXA data, and shows that compared with the participants in this study, those

excluded due to missing bone data had lower socioeconomic position and had slightly lower birthweight and slightly higher BMI (eTable 2). Those missing data on model covariates were also excluded from the analysis.

eTable 1. Comparison on Model Covariates Between Participants Included in the Linear Spline Models That Had All Six DXA Measures With Those Also Included in the Analysis But Were Missing at Least One DXA Measure

	Males			Females	
	Included in analyses: all six DXA measures available (n=585)	Included in analyses: missing ≥1 DXA measure (n=2608)		Included in analyses: all six DXA measures available (n=993)	Included in analyses: missing ≥1 DXA measure (n=2203)
Birth weight – g [mean (SD)]	3484.1 (548.2)	3472.0 (597.8)		3375.3 (499.7)	3376.1 (509.3)
Ethnicity [No. (%)]					
White	575 (98.3)	2562 (98.2)		972 (97.9)	2164 (98.2)
Non-white	10 (1.7)	46 (1.8)		21 (2.1)	39 (1.8)
Maternal education [No. (%)]					
Certificate of Secondary Education	37 (6.3)	325 (12.5)		58 (5.8)	328 (14.9)
Vocational	34 (5.8)	248 (9.5)		54 (5.4)	202 (9.2)
General Certificate of Education: Ordinary Level	175 (29.9)	985 (37.8)		343 (34.5)	764 (34.7)
General Certificate of Education: Advanced Level	179 (30.6)	683 (26.2)		310 (31.2)	581 (26.4)
Degree or higher	160 (27.4)	367 (14.1)		228 (23.0)	328 (14.9)
BMI at age 7y – kg/m^2 [mean (SD)]	15.8 (1.7)	16.1 (1.9)		16.0 (1.8)	16.4 (2.2)
Daily energy intake at age 7y– kJ/day [mean (SD)]	7712.9 (1603.2)	7801.7 (1935.2)		7431.4 (1612.6)	7498.5 (1821.7)
APHV– years [mean (SD)]	13.4 (0.9)	13.5 (0.9)		11.7 (0.8)	11.6 (0.8)

© 2019 Elhakeem A et al. *JAMA Network Open.*

eTable 2. Comparison on Model Covariates Between Those Included in the Linear Spline Models With Those That Were Excluded From the Analysis Due to Missing All Six DXA Measures, Despite Having Data on APHV

	Males			Females	
	Included in linear spline models (n=3193)	Excluded due to missing all DXA (n=851)		Included in linear spline models (n=3196)	Excluded due to missing all DXA (n=755)
Birth weight – g [mean (SD)]	3474.2 (574.0)	3449.4 (578.1)		3375.8 (506.3)	3372.7 (506.3)
Ethnicity [No. (%)]					
White	3137 (98.3)	727 (96.7)		3136 (98.1)	621 (97.8)
Non-white	56 (1.8)	25 (3.3)		60 (1.9)	14 (2.2)
Maternal education [No. (%)]					
Certificate of Secondary Education	362 (11.3)	161 (21.3)		386 (12.1)	172 (26.8)
Vocational	282 (8.8)	74 (9.8)		256 (8.0)	75 (11.7)
General Certificate of Education: Ordinary Level	1160 (36.3)	266 (35.2)		1107 (34.6)	217 (33.8)
General Certificate of Education: Advanced Level	862 (27.0)	157 (20.8)		891 (27.8)	113 (17.6)
Degree or higher	527 (16.5)	97 (12.9)		556 (17.4)	66 (10.3)
BMI at age 7y – kg/m^2 [mean (SD)]	16.1 (1.9)	16.2 (1.9)		16.3 (2.1)	16.8 (2.9)
Daily energy intake at age 7y– kJ/day [mean (SD)]	7785.5 (1878.8)	7731.9 (2068.8)		7477.7 (1759.8)	7459.9 (2029.1)
APHV– years [mean (SD)]	13.5 (0.9)	13.5 (0.7)		11.6 (0.8)	11.7 (0.8)

© 2019 Elhakeem A et al. *JAMA Network Open.*

eReferences

1. Cole TJ, Donaldson MD, Ben-Shlomo Y. SITAR--a useful instrument for growth curve analysis. Int J Epidemiol. 2010;39(6):1558-66.
2. Cole TJ, Kuh D, Johnson W, Ward KA, Howe LD, Adams JE, et al. Using Super-Imposition by Translation And Rotation (SITAR) to relate pubertal growth to bone health in later life: the Medical Research Council (MRC) National Survey of Health and Development. Int J Epidemiol. 2016.
3. Frysz M, Howe L, Tobias J, Paternoster L. Using SITAR (SuperImposition by Translation and Rotation) to estimate age at peak height velocity in Avon Longitudinal Study of Parents and Children. Wellcome Open Res; 2018.
4. Mahmoud O, Granell R, Tilling K, Minelli C, Garcia-Aymerich J. Association of Height Growth in Puberty with Lung Function: A Longitudinal Study. Am J Respir Crit Care Med. 2018;doi: 10.1164/rccm.201802-0274OC
5. Tilling K, Macdonald-Wallis C, Lawlor DA, Hughes RA, Howe LD. Modelling childhood growth using fractional polynomials and linear splines. Annals of nutrition & metabolism. 2014;65(2-3):129-38.
6. Howe LD, Tilling K, Matijasevich A, Petherick ES, Santos AC, Fairley L, et al. Linear spline multilevel models for summarising childhood growth trajectories: A guide to their application using examples from five birth cohorts. Statistical methods in medical research. 2016;25(5):1854-74.

© 2019 Elhakeem A et al. *JAMA Network Open.*

eTable 3. Gains in BMD and BMC During Each Period (A) From Childhood (Prepuberty) to Adulthood, With Age Centered at Pubertal Age (ie, APHV) and (B) From Puberty to Adulthood Per 1-Year Older Age at Puberty

	BMD – *g/cm²/yr.* (95% CI)		BMC – *g/yr.* (95% CI)	
	Males	Females	Males	Females
*A. Rates relative to APHV (n=6389)**				
-7.7 to -0.9 yrs.	0.054 (0.047 to 0.062)	0.014 (0.000 to 0.028)	289.109 (256.887 to 321.331)	74.065 (5.229 to 139.537)
-0.9 to 2.1 yrs	0.137 (0.125 to 0.150)	0.106 (0.098 to 0.115)	644.480 (592.822 to 696.138)	428.786 (390.064 to 463.701)
2.1 to 3.7 yrs.	0.078 (0.060 to 0.096)	0.047 (0.037 to 0.058)	456.124 (378.401 to 533.847)	312.841 (263.411 to 306.351)
3.7 to 16.0 yrs.	0.013 (0.005 to 0.021)	0.005 (0.001 to 0.009)	-35.423 (-69.913 to -0.934)	16.057 (-2.495 to 34.609)
*B. Rates per 1-year older APHV (n=5477)***				
up to 14 yrs	0.012 (0.003 to 0.020)	0.010 (0.009 to 0.011)	92.259 (49.562 to 135.955)	40.902 (35.538 to 46.265)
14 to 16 yrs	0.013 (0.011 to 0.015)	0.014 (0.014 to 0.015)	85.125 (76.005 to 94.244)	61.378 (56.995 to 65.761)
16 to 18 yrs	0.011 (0.010 to 0.013)	0.003 (0.003 to 0.004)	64.252 (58.846 to 69.658)	19.374 (15.398 to 23.350)
18 to 25 yrs	0.002 (0.001 to 0.003)	0.000 (-0.001 to 0.000)	13.368 (10.823 to 15.913)	5.793 (3.902 to 7.683)

* Knots at the 12, 14, 16 year assessments. Age centred at APHV (i.e. 0=APHV): models includes APHV:age splines interaction terms, and adjsutment for birth weight, maternal education, childhood BMI and diet.

** Knots at the 14, 16 and 18 year assessments. Models includes APHV:age splines interaction terms, and adjsutment for birth weight, ethnicity, maternal education and childhood BMI and diet. These models excluded bone measures recorded before APHV.

© 2019 Elhakeem A et al. *JAMA Network Open.*

eFigure 1 Observed Whole-body BMD and BMC at Each Follow-up Assessment from 10 to 25 Years

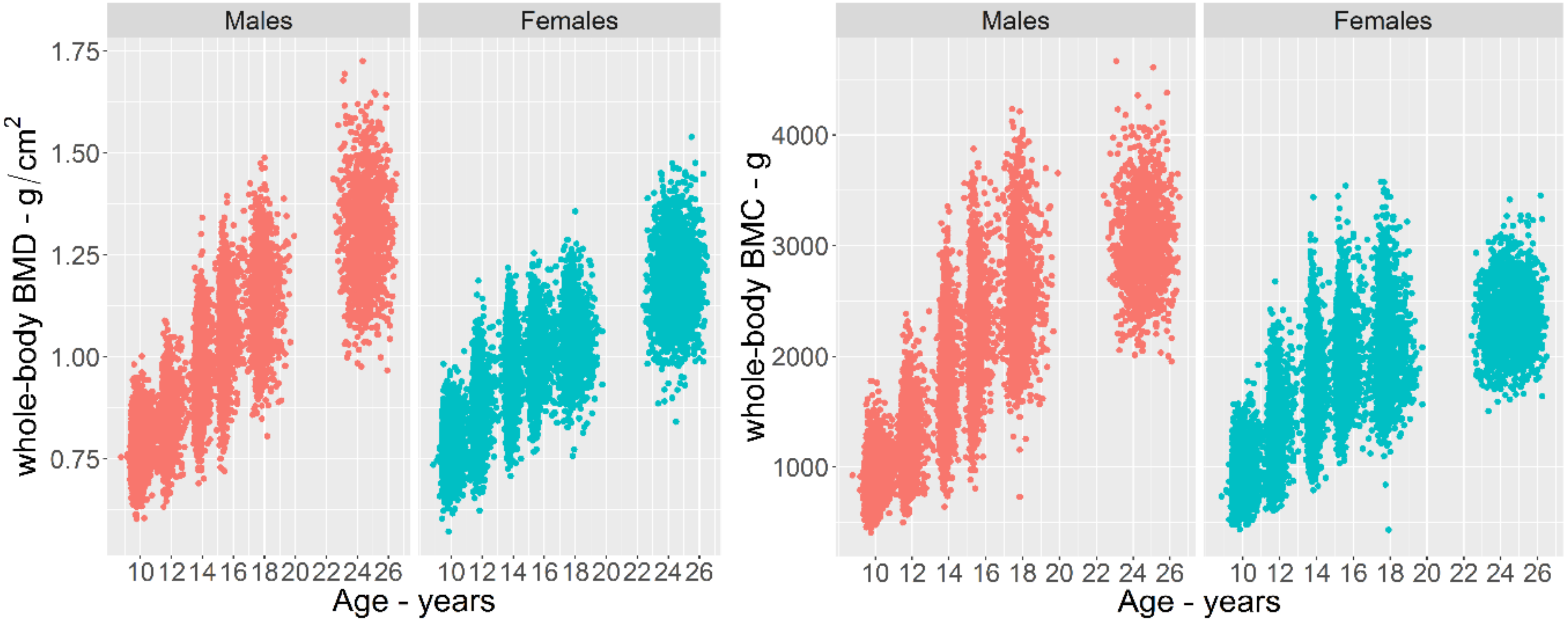

© 2019 Elhakeem A et al. *JAMA Network Open.*

eFigure 2 Predicted Mean Trajectories of Whole-body BMD and BMC From APHV to Adulthood for Individuals in the 10th, 50th, and 90th APHV Percentiles, Using APHV From Measured Heights (Dotted points represent predicted values.)

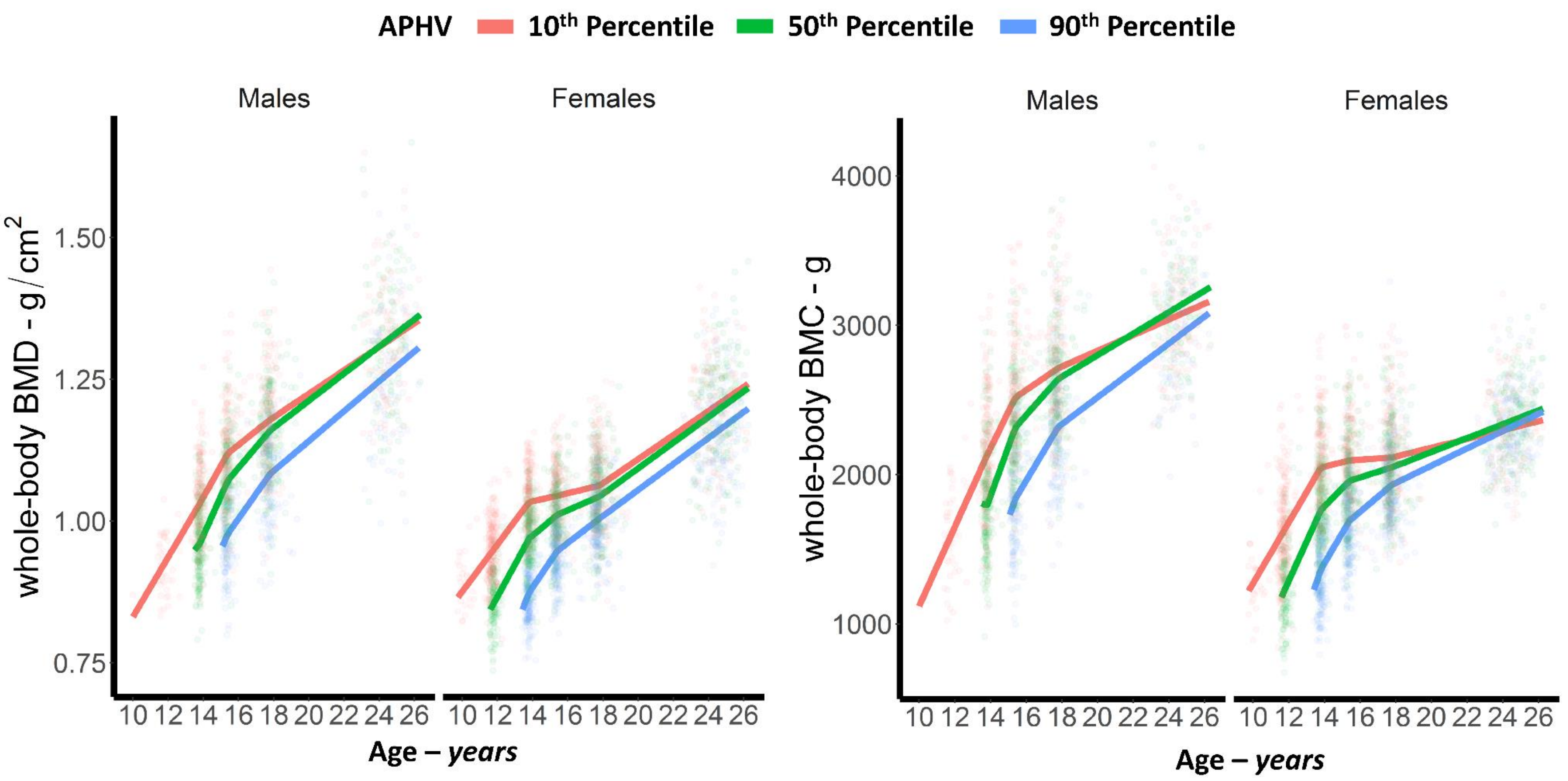

© 2019 Elhakeem A et al. *JAMA Network Open.*

eFigure 3. Predicted Mean Trajectories of Whole-body BMD and BMC From Menarche to Adulthood for Females in the 10th, 50th, and 90th Age at Menarche Percentiles (Dotted points represent predicted values.)

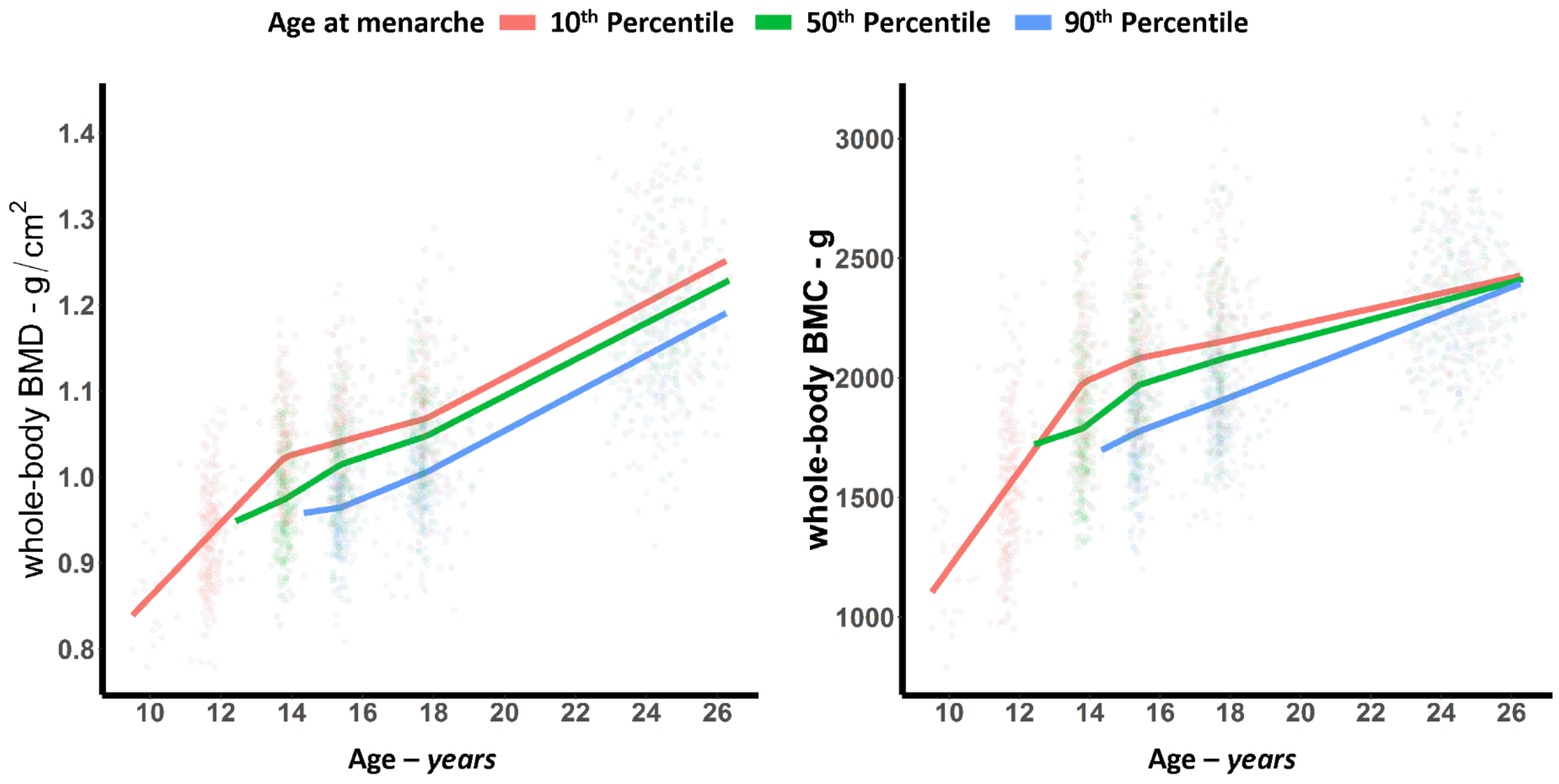

© 2019 Elhakeem A et al. *JAMA Network Open.*

eFigure 4. Predicted Mean Trajectories of Arms BMD and BMC From APHV to Adulthood for Individuals in the 10th, 50th, and 90th APHV Percentiles (Dotted points represent predicted values)

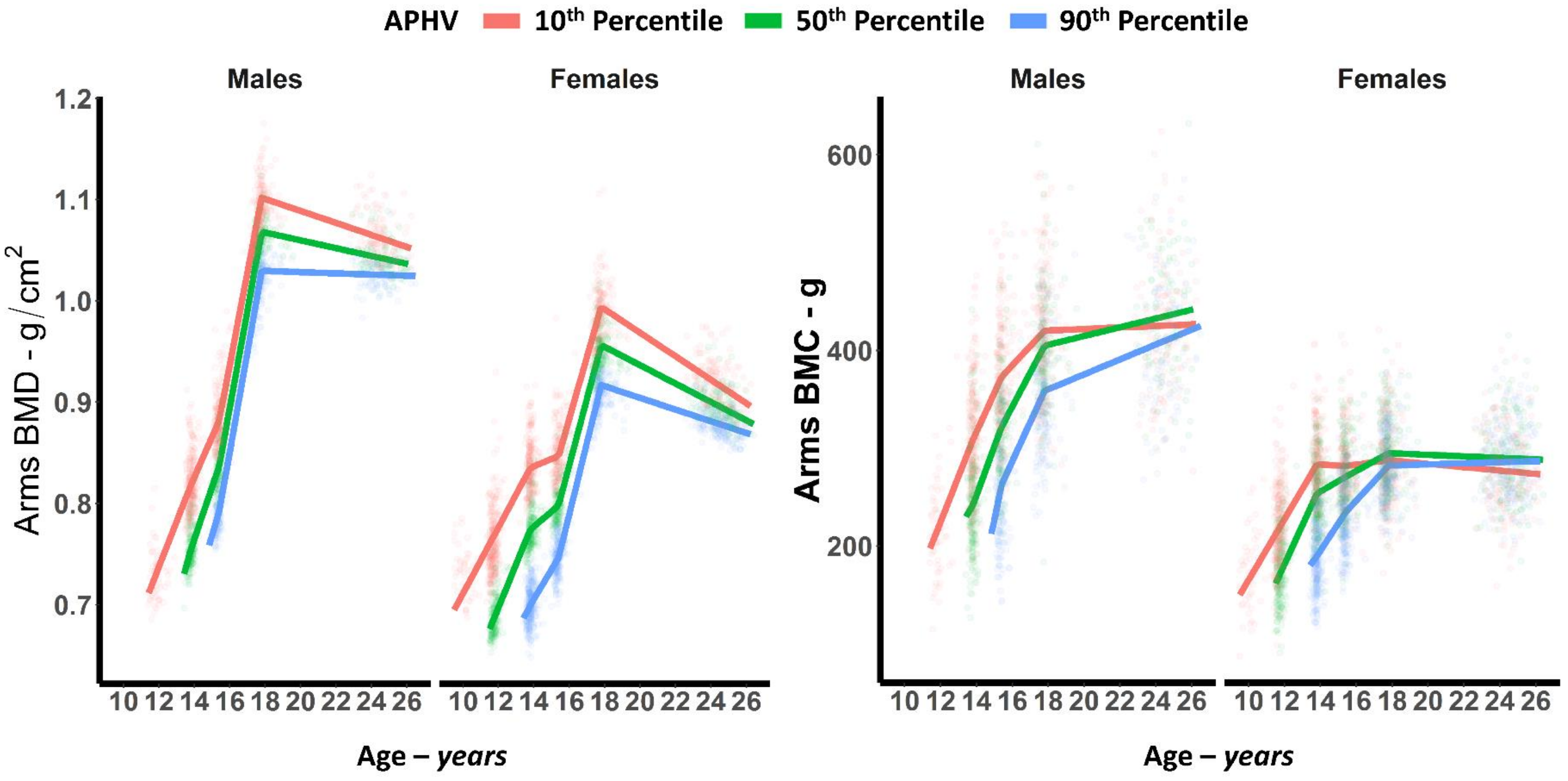

© 2019 Elhakeem A et al. *JAMA Network Open.*

eFigure 5. Predicted Mean Trajectories of Legs BMD and BMC From APHV to Adulthood for Individuals in the 10th, 50th, and 90th APHV Percentiles (Dotted points represent predicted values.)

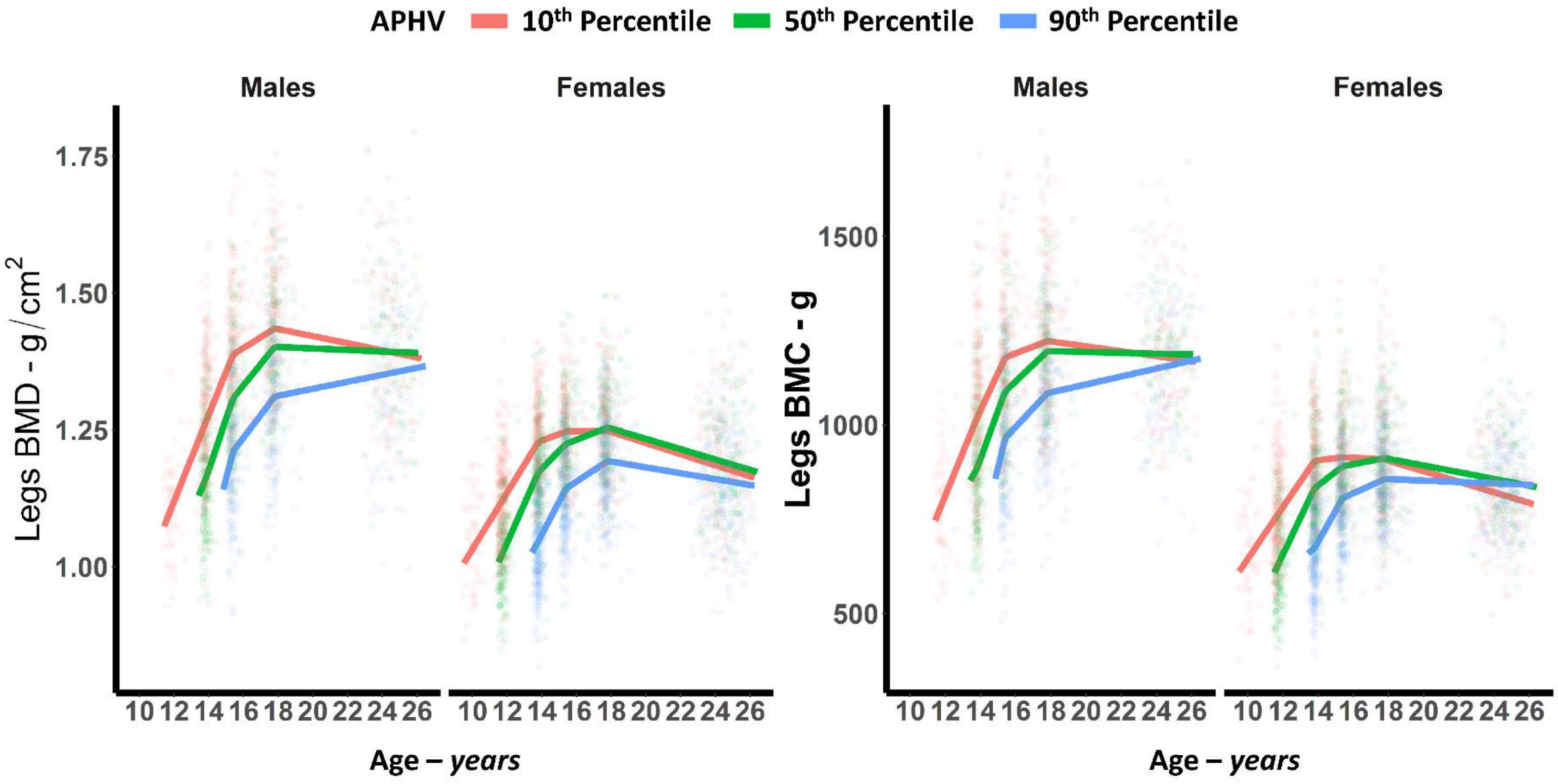

© 2019 Elhakeem A et al. *JAMA Network Open.*

eFigure 6. Predicted Mean Trajectories of Trunk BMD and BMC From APHV to Adulthood for Individuals in the 10th, 50th, and 90th APHV Percentiles (Dotted points represent predicted values.)

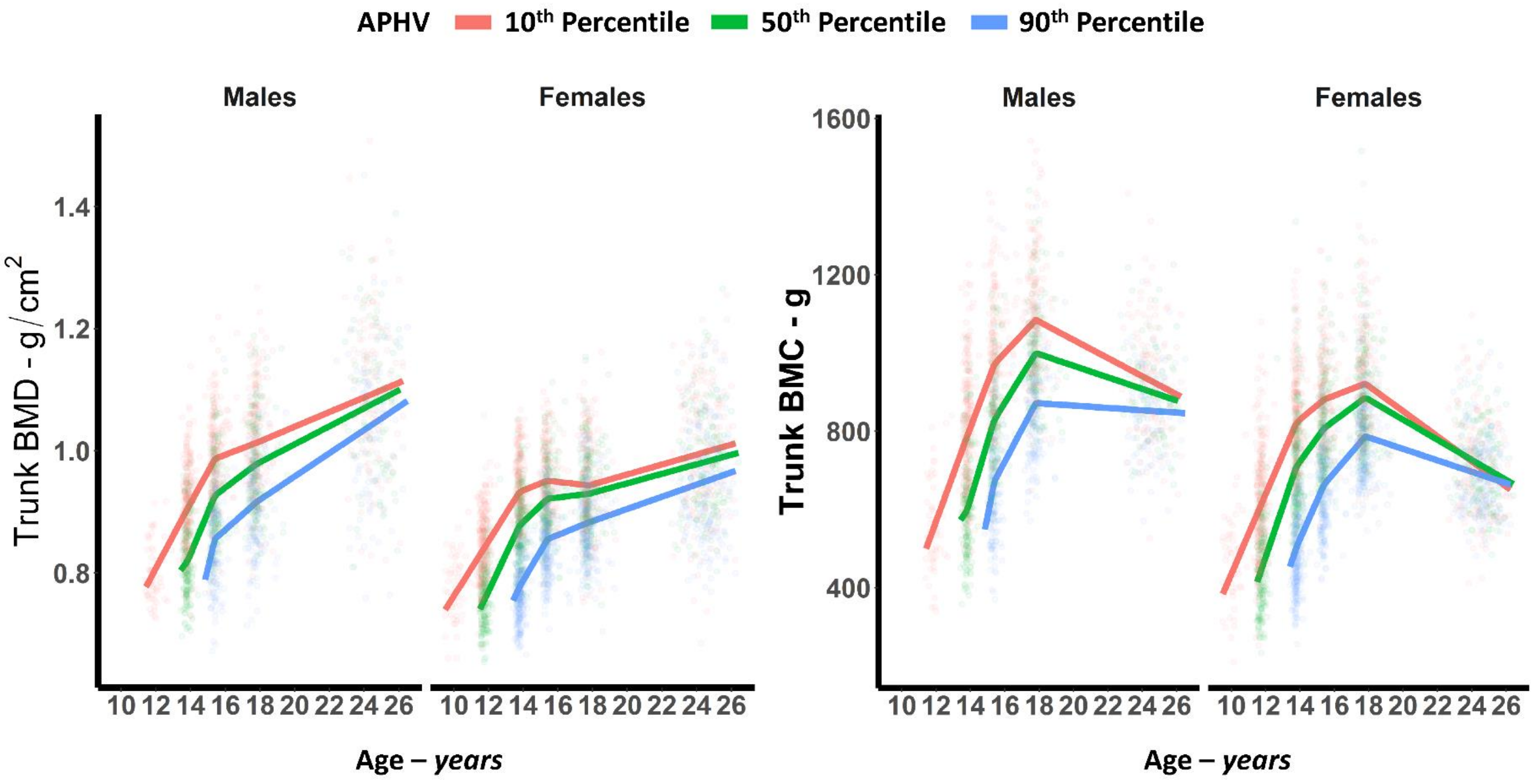

© 2019 Elhakeem A et al. *JAMA Network Open.*

eFigure 7. Predicted Mean Trajectories of Spine BMD and BMC From APHV to Adulthood for Individuals in the 10th, 50th, and 90th APHV Percentiles (Dotted points represent predicted values.)

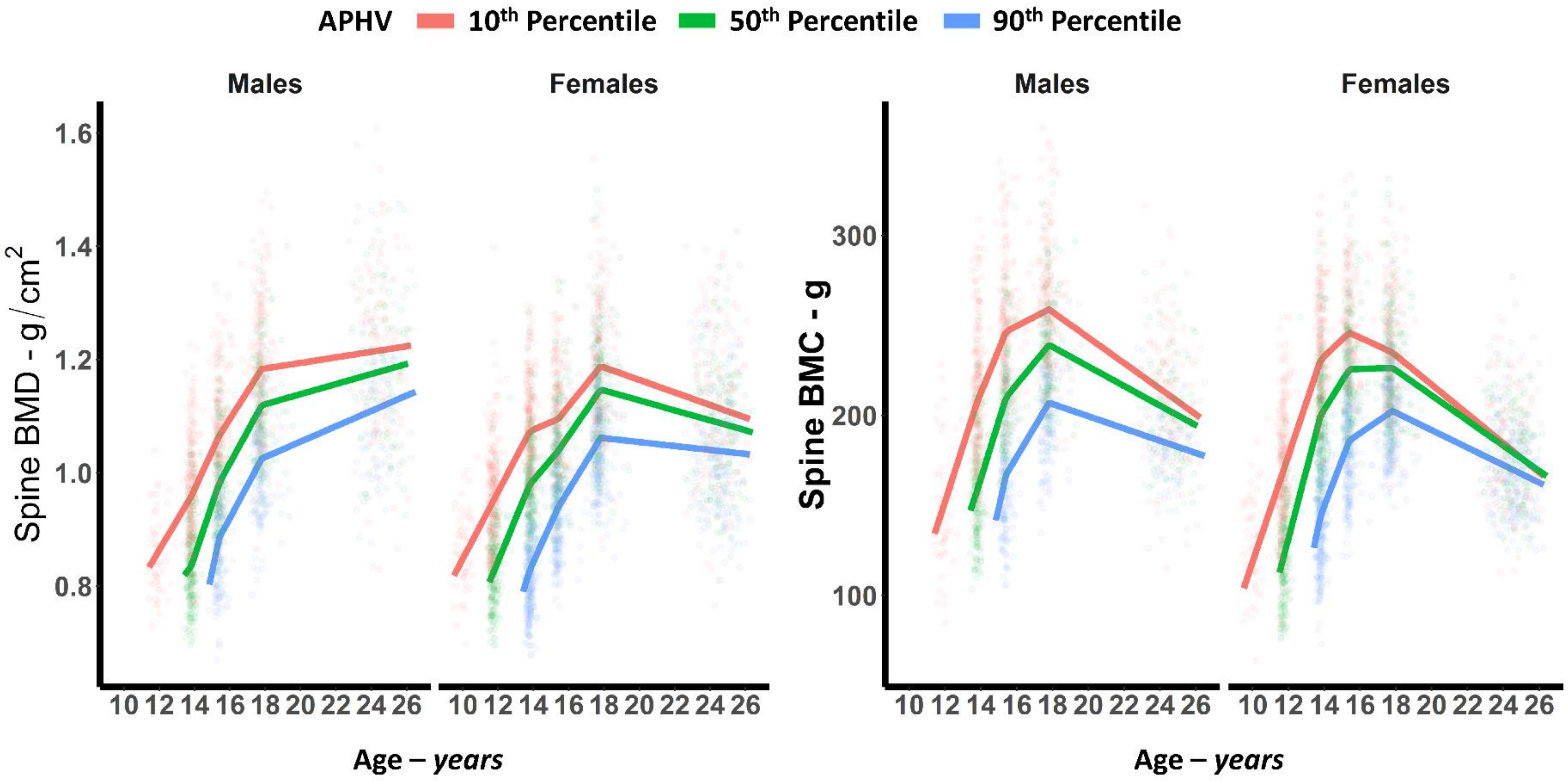

© 2019 Elhakeem A et al. *JAMA Network Open.*

eFigure 8. Predicted Mean Trajectories of Ribs BMD and BMC From APHV to Adulthood for IIndividuals in the 10th, 50th, and 90th APHV Percentiles (Dotted points represent predicted values.)

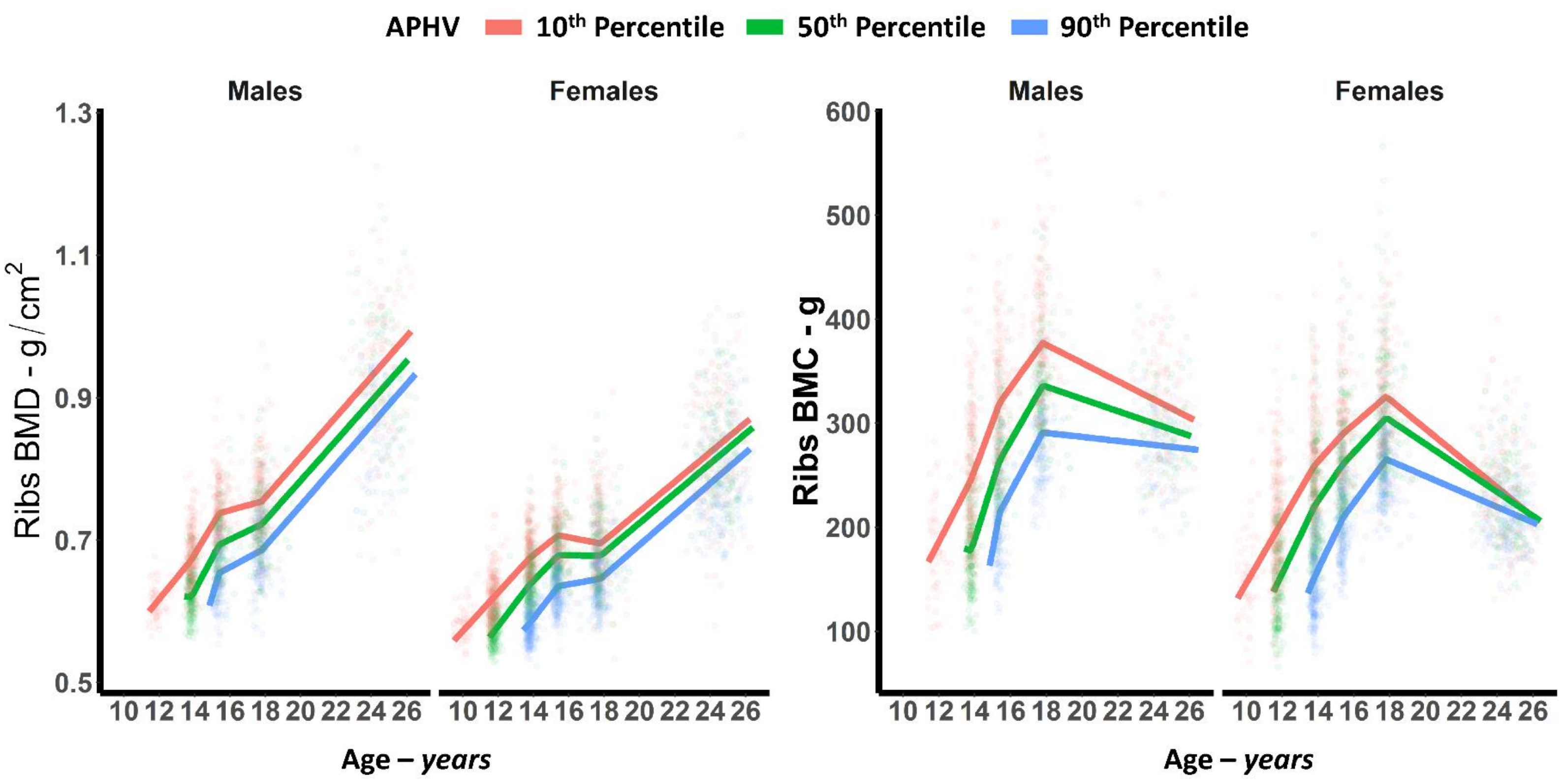

© 2019 Elhakeem A et al. *JAMA Network Open.*

eFigure 9. Predicted Mean Trajectories of Pelvis BMD and BMC From APHV to Adulthood for Individuals in the 10th, 50th, and 90th APHV Percentiles (Dotted points represent predicted values.)

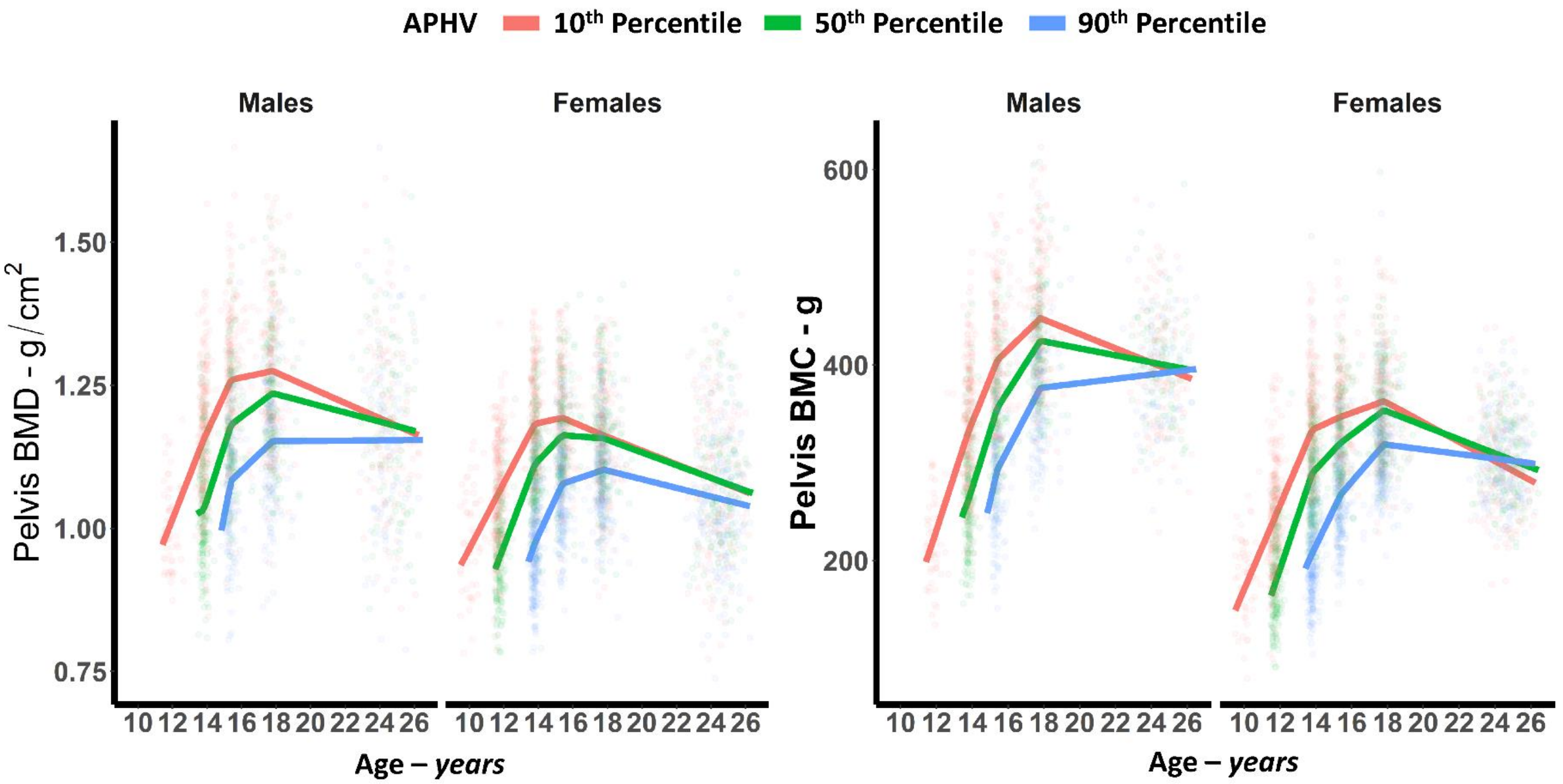

© 2019 Elhakeem A et al. *JAMA Network Open.*

eFigure 10. Predicted Mean Trajectories of Total Hip and Femur Neck BMD From APHV to Adulthood for Individuals in the 10th, 50th, and 90th APHV Percentiles

(Dotted points represent predicted values.)

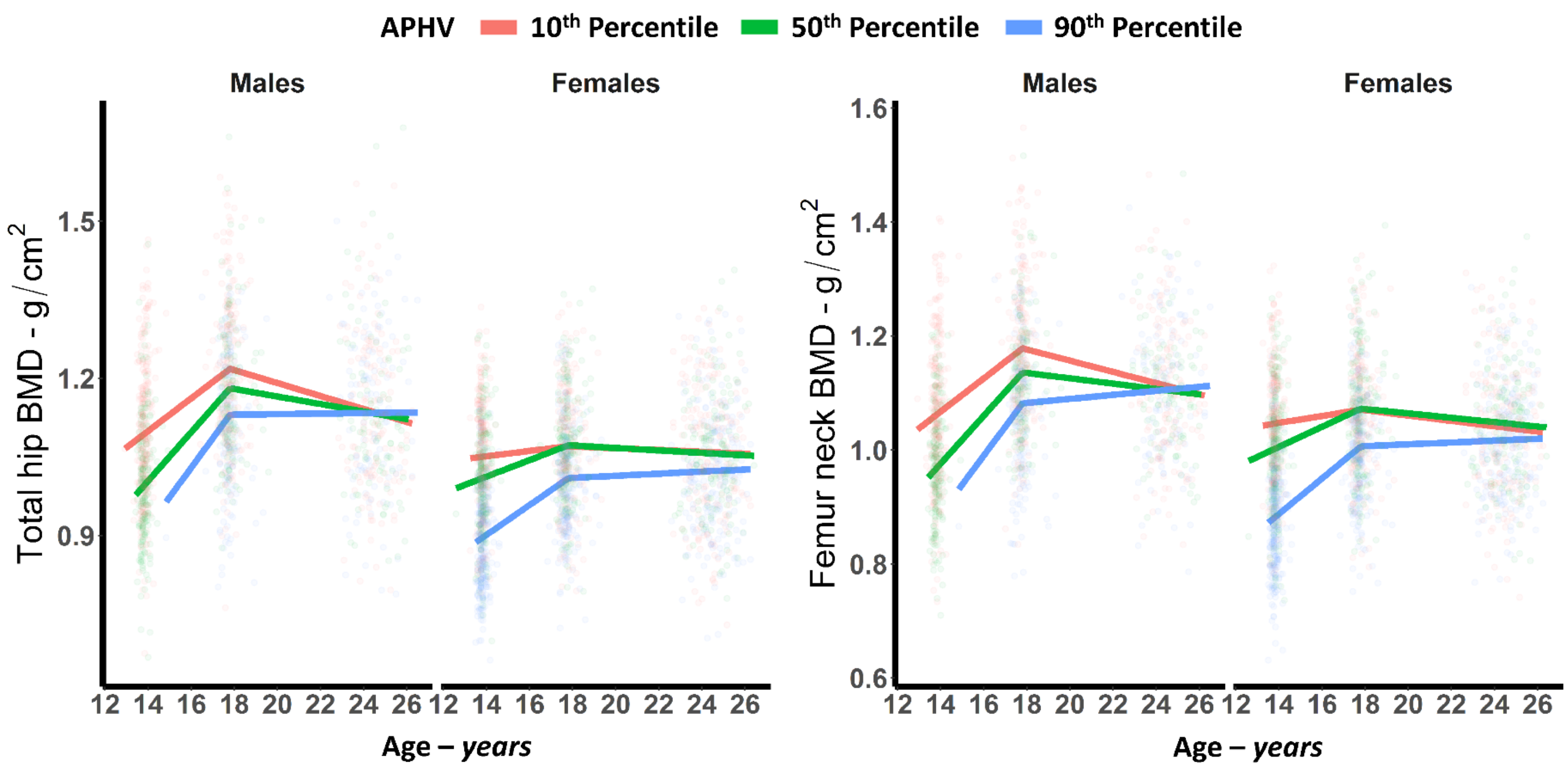

© 2019 Elhakeem A et al. *JAMA Network Open.*
